# Supplementary material for: Long-term relapse-free survival enabled by integrating targeted antibacteria in antitumor treatment
Source: Nat Commun. 2024 May 17;15:4194. doi: 10.1038/s41467-024-48662-x (PMC11101653; doi:10.1038/s41467-024-48662-x)
Supplement: Supplementary file 1 — Supplementary Information [file 41467_2024_48662_MOESM1_ESM.pdf]

Supplementary Information

Long-term relapse-free survival enabled by integrating targeted antibacteria in antitumor treatment

Yuanlin Wang[1], Yaqian Han[2], Chenhui Yang[1], Tiancheng Bai[1], Chenggang Zhang[2], Zhaotong Wang[2], Ye Sun[2,*], Ying Hu[3], Flemming Besenbacher[4], Chunying Chen[5], and Miao Yu[1,*]

1. School of Chemistry and Chemical Engineering, Harbin Institute of Technology, Harbin 150001, China

2. School of Instrumentation Science and Technology, Harbin Institute of Technology, Harbin 150001, China

3. School of Life Science and Technology, Harbin Institute of Technology, Harbin 150001, China

4. Interdisciplinary Nanoscience Center (iNANO), Aarhus University, Aarhus 8000, Denmark

5. National Center for Nanoscience and Technology, Chinese Academy of Sciences, Beijing 100190, China

Correspondence to: miaoyu_che@hit.edu.cn, sunye@hit.edu.cn

Supplementary Information includes:

Supplementary Figs. 1–46;

Supplementary Tables 1 and 2.

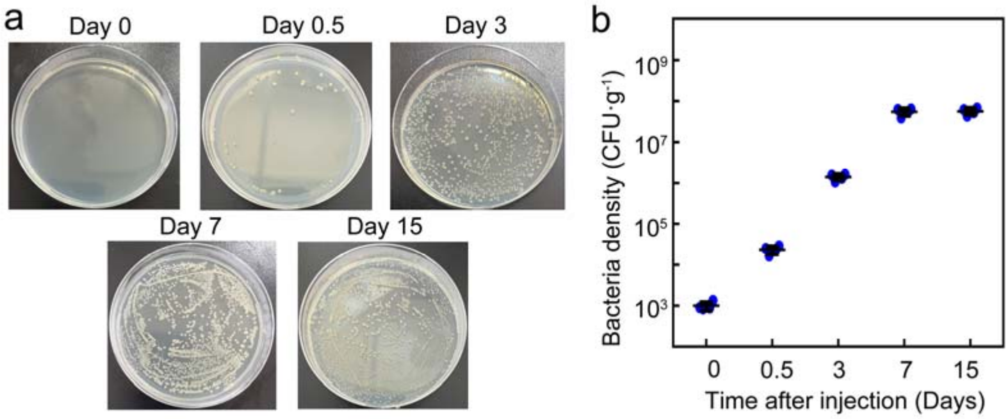

Supplementary Fig. 1 | a, Representative pictures showing bacteria culture analysis for the infected breast tumors of EC Group before (Day 0) and after injection with *Escherichia coli* (*E. coil*). All experiments are conducted at least twice independently, yielding similar results. **b,** Bacteria density measured from the breast tumors of EC group before and at the various time points after injection of *E. coli* (*n* = 4 mice per group).

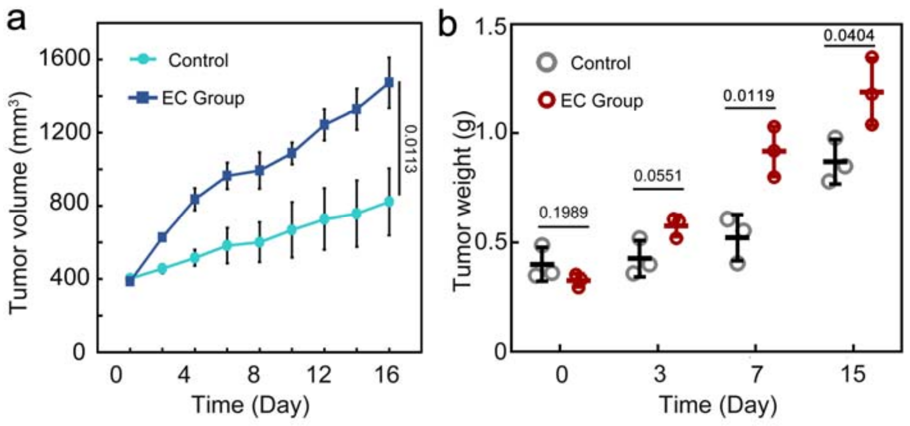

Supplementary Fig. 2 | a, Tumor volume (n = 4 mice per group) and **b,** mean tumor weight (n = 3 mice per group) of *E. coli*-infected group, compared with the non-infected mice group (Control). Data are presented as mean ± standard deviation. One-way ANOVA with Tukey's post hoc test (**b**) or two-tailed Student's *t*-test (**a**). $P < 0.05$ is considered to be statistically significant.

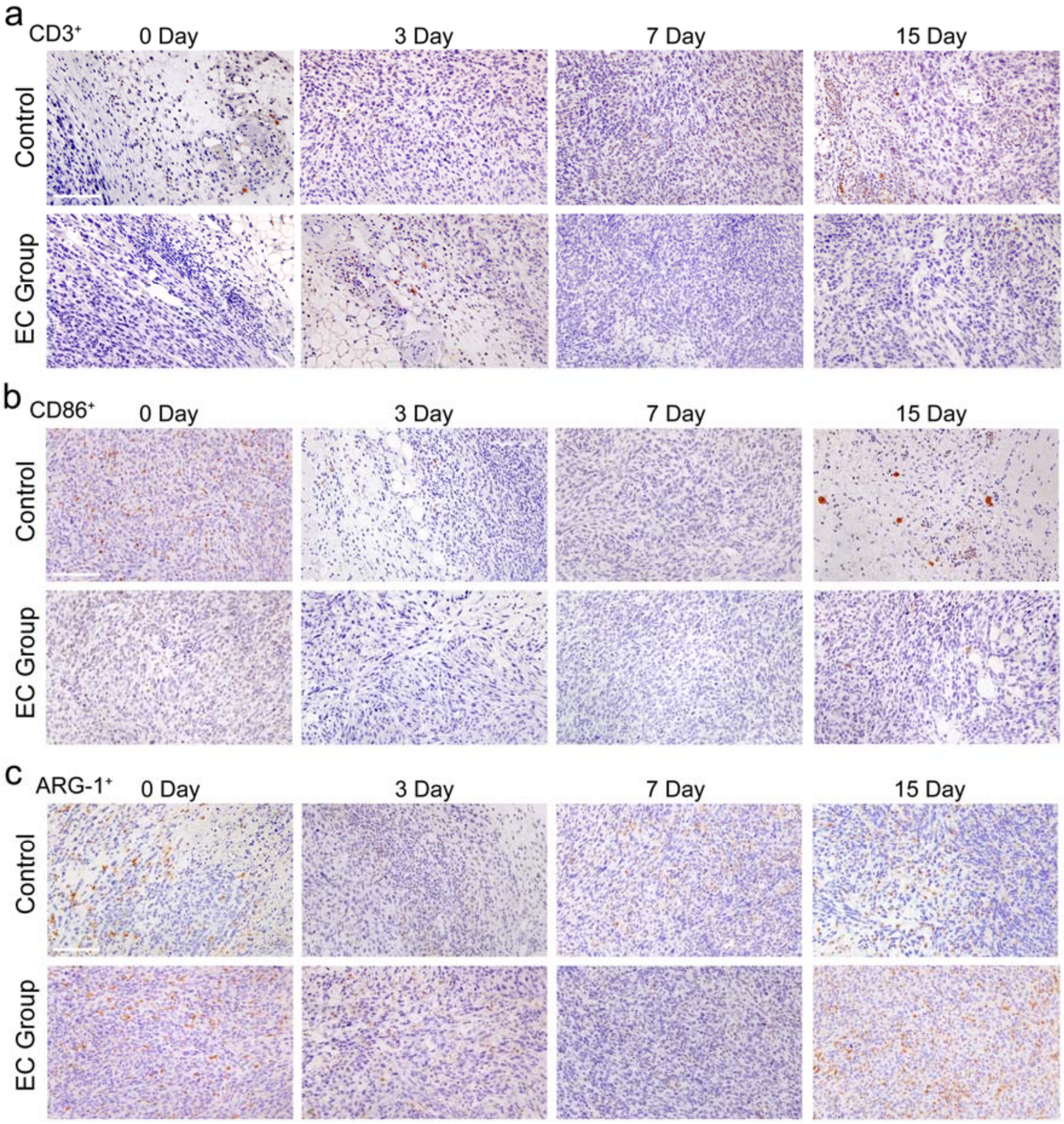

Supplementary Fig. 3 | Representative images of immunohistochemical analysis for tumor tissues stained with **a,** $CD3^+$ to evaluate T cells, **b,** $CD86^+$ to evaluate M1-like tumor-associated macrophages (TAMs) and dendritic cells, and **c,** ARG-1^+ to evaluate M2-like TAMs (Scale bar = 100 μm). All experiments are conducted at least twice independently, yielding similar results.

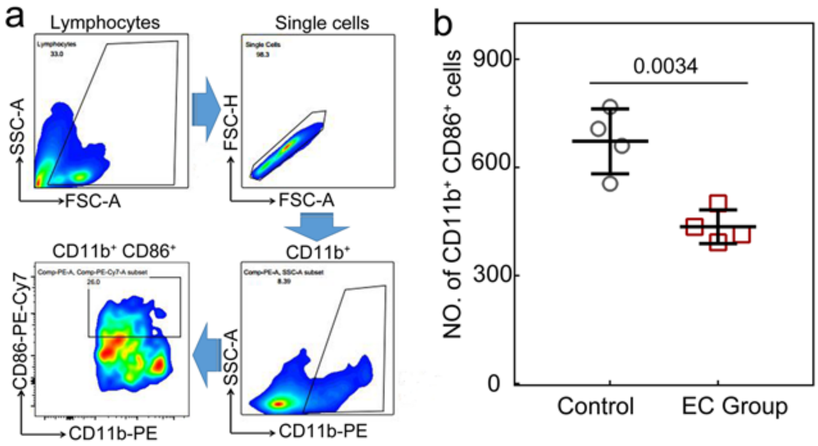

Supplementary Fig. 4 | a, Flow cytometric gating strategy for M1-like TAMs (CD11b$^+$, CD86$^+$) panel of Control and EC. **b,** The number of CD11b$^+$ and CD86$^+$ M1-like TAMs per 50,000 cells of tumors in Control and EC (n = 4 mice per group). Data are presented as mean ± standard deviation. One-way ANOVA with Tukey's post hoc test. $P < 0.05$ is considered to be statistically significant.

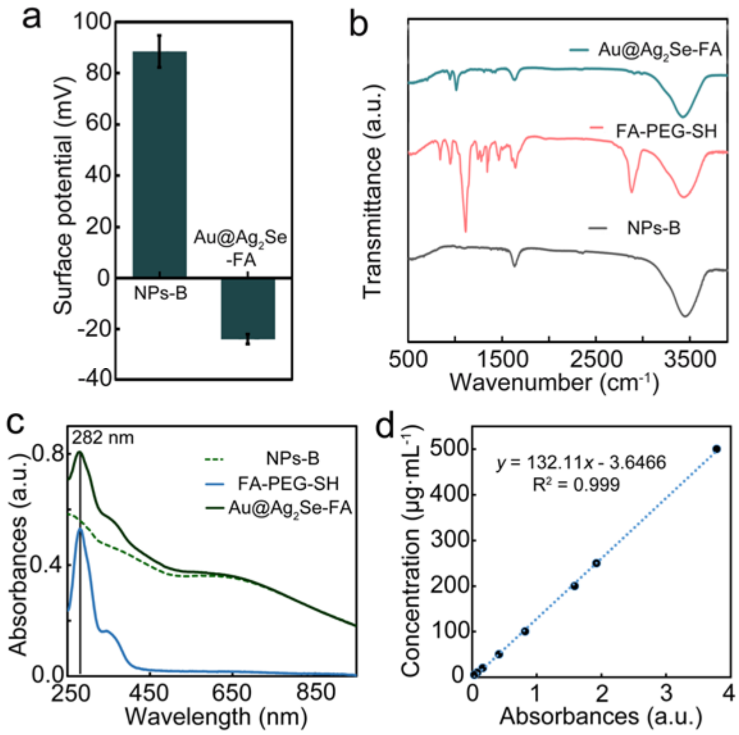

Supplementary Fig. 5 | a, Surface potential, **b,** Fourier transform infrared spectra, and **c,** UV-*vis*-NIR spectra of Au@Ag_2Se before (bare NPs, NPs-B) and after reaction with folic acid-polyethylene glycol-thiol (FA-PEG-SH) (Au@Ag_2Se-FA). **d,** Absorbance of FA-PEG-SH dispersions at 282 nm and various concentrations. According to **c** and **d**, the FA-PEG-SH load on 100 μg Au@Ag_2Se-FA is estimated to be ~ 29 μg (~3.8 μg of FA). All experiments are conducted at least twice independently, yielding similar results.

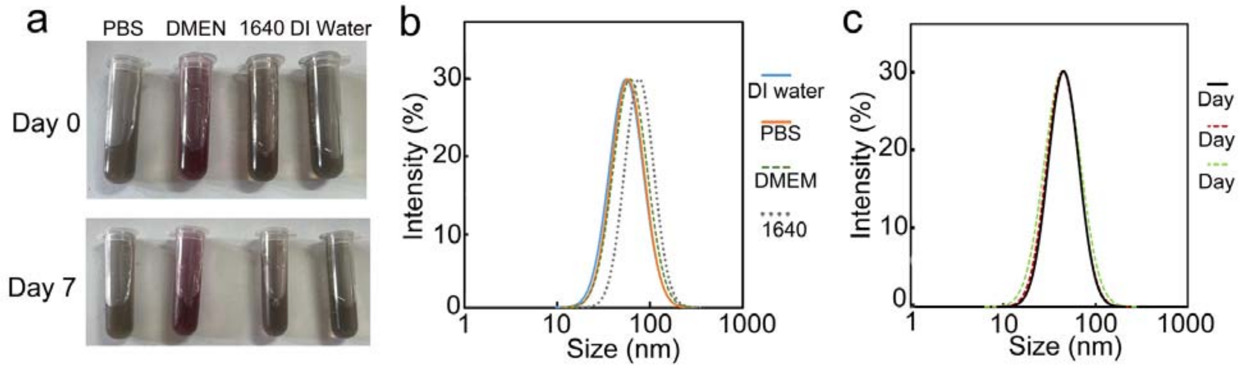

Supplementary Fig. 6 | a, Digital photos of Au@Ag_2Se-FA dispersed in various physiological solutions, including phosphate buffer saline (PBS), Dulbecco's modified Eagle's medium with 10% FBS (DMEM), RPMI medium 1640 basic with 10% FBS (1640), and DI water after storing at room temperature for seven days. **b,** Hydrodynamic size of Au@Ag_2Se-FA NPs dispersed in DI water, PBS, DMEM, and 1640, and **c,** Au@Ag_2Se-FA dispersed in DI water stored at room temperature for varied durations. All experiments are conducted at least twice independently, yielding similar results.

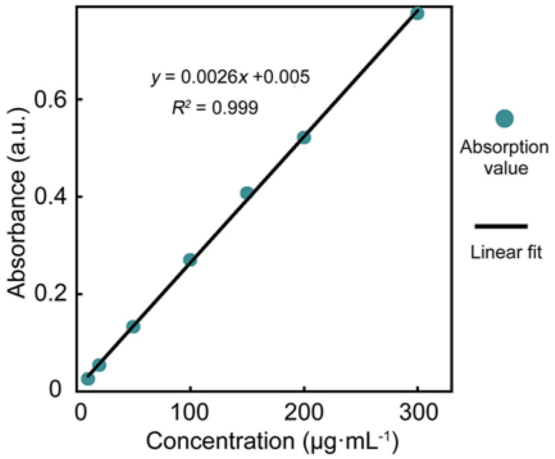

Supplementary Fig. 7 | Absorbance of Au@Ag_2Se-FA dispersions at 808 nm and different dispersion concentrations. All experiments are conducted at least twice independently, yielding similar results.

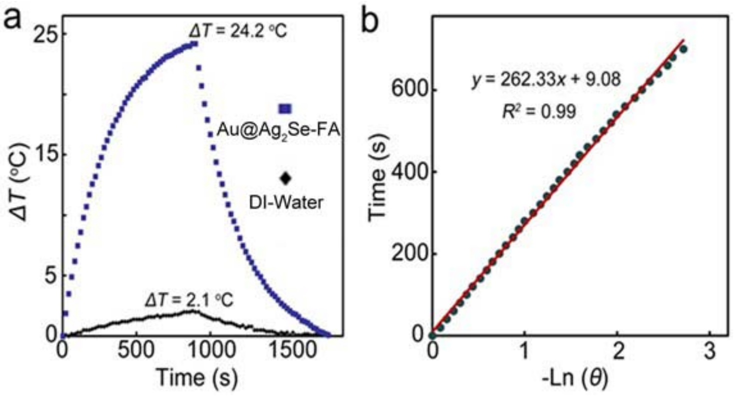

Supplementary Fig. 8 | a, Heating-cooling curve of Au@Ag_2Se-FA dispersion (100 μg·mL^{-1}) upon 808 nm irradiation (1.2 W·cm^{-2}), compared with that of DI water. **b,** Linear fitting between the system cooling time and the negative natural logarithm of the temperature driving force, where the time constant of the heat transfer is determined to be τ_s = 262.33 s. All experiments are conducted at least twice independently, yielding similar results.

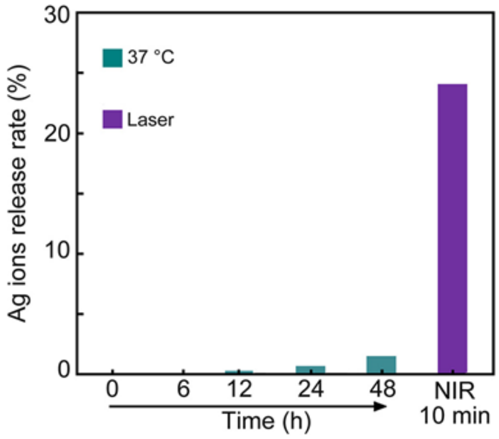

Supplementary Fig. 9 | Release of Ag ions from Au@Ag_2Se-FA at different conditions measured by inductively coupled plasma mass spectroscopy (ICP-MS), showing an Ag^+ release rate < 1.5% after incubated at 37 °C for 48 h and a release rate > 24.1% upon 808 nm irradiation (1.2 W·cm^{-2}) for 10 min. All experiments are conducted at least twice independently, yielding similar results.

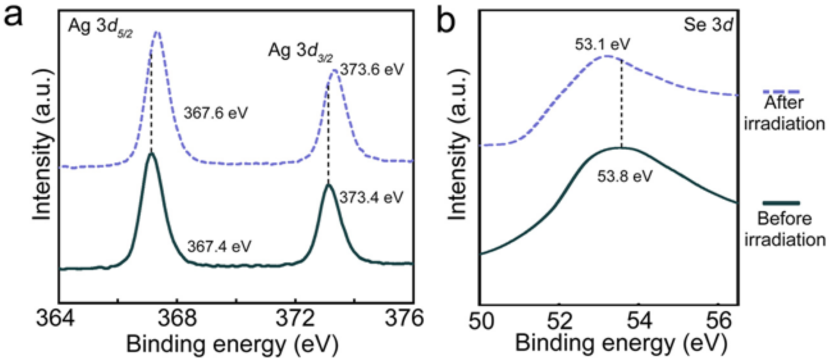

Supplementary Fig. 10 | a, Ag 3*d* and **b,** Se 3*d* X-ray photoelectron spectra of Au@Ag_2Se-FA before and after 808 nm irradiation (1.2 W·cm^{-2}, 10 min). After irradiation, both peaks in the Ag *3d* spectrum have a red shift of 0.2 eV and the peak in the Se *3d* spectrum has a blue shift of 0.7 eV, compared with the peaks of Au@Ag_2Se-FA prior to irradiation. The variations are attributed to the Ag vacancies induced by the formation of Ag nanocrystals upon irradiation, and the Ag_2Se shell is modified to be $Ag_{2-x}Se$. All experiments are conducted at least twice independently, yielding similar results.

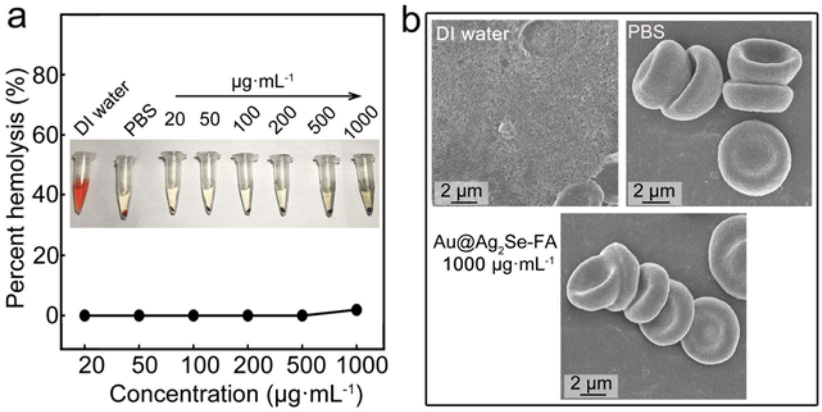

Supplementary Fig. 11 | a, Hemolytic percentage and digital image of red blood cells (RBCs) incubated with Au@Ag_2Se-FA dispersions at different concentrations (20–1000 μg·mL^{-1}) at 37 °C for 24 h, with the cells incubated with PBS and DI water as a negative and positive control, respectively. The hemolysis percentage is < 1.9% even for the RBCs incubated with a high Au@Ag_2Se-FA concentration of 1000 μg·mL^{-1}. **b,** SEM images of RBCs treated with the Au@Ag_2Se-FA dispersion (1000 μg·mL^{-1}), DI water, and PBS, respectively. The cells are severely destroyed in DI water; RBCs treated by the Au@Ag_2Se-FA dispersions show no noticeable variation from those treated with PBS. All experiments are conducted at least twice independently, yielding similar results.

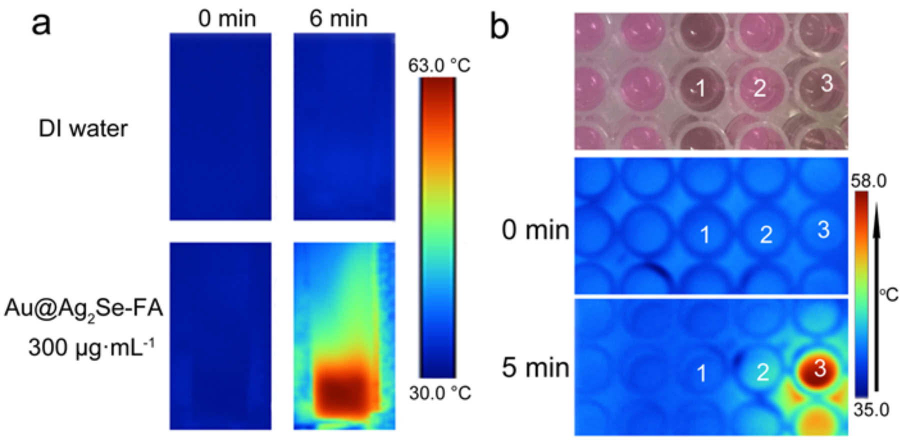

Supplementary Fig. 12 | a, Infrared thermal (IRT) image of Au@Ag_2Se-FA dispersion (300 µg·mL^{-1}) upon NIR irradiation (808 nm, 1.2 W·cm^{-2}). **b,** Digital and IRT images of cells before and upon irradiation for 5 min. 4T1 cells incubated with the Au@Ag_2Se-FA dispersion (100 µg·mL^{-1}) are in Well #1 and #3; cells in pure culture medium are in Well #2; Well #2 and #3 are irradiated. All experiments are conducted at least twice independently, yielding similar results.

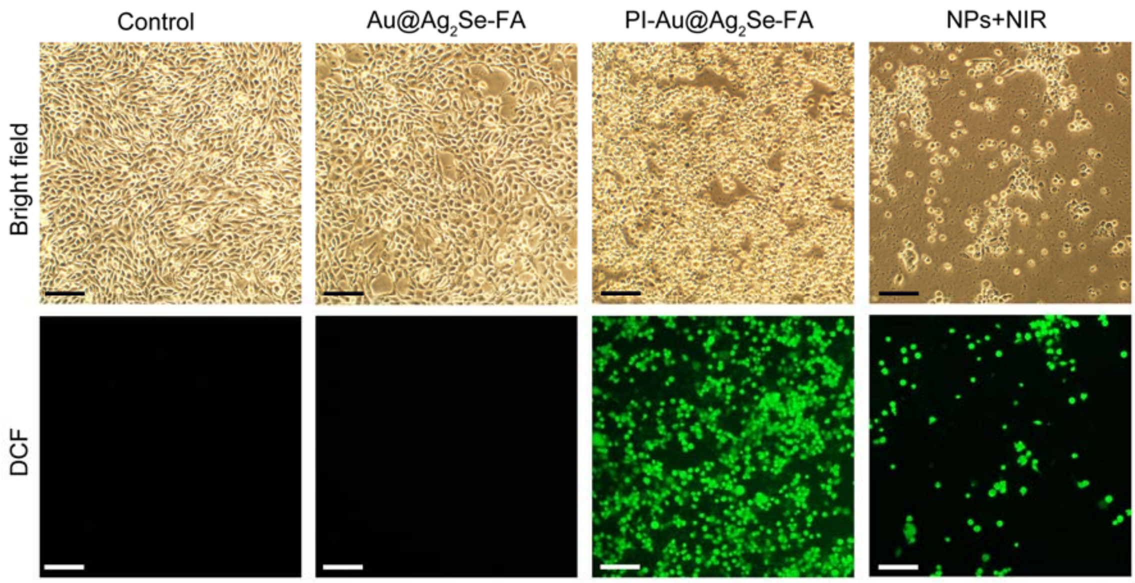

Supplementary Fig. 13 | Fluorescence images of the 2,7-dichlorodihydrofluorescein diacetate-stained 4T1 cells after 24 h incubation with i) $Au@Ag_2Se$-FA (100 μg·mL^{-1}) without irradiation, ii) $Au@Ag_2Se$-FA (100 μg·mL^{-1}) pre-irradiated by NIR laser (808 nm, 0.8 W·cm^{-2}, 10 min, PI-$Au@Ag_2Se$-FA) without irradiation afterwards, and iii) $Au@Ag_2Se$-FA (100 μg·mL^{-1}) plus NIR laser (808 nm, 0.8 W·cm^{-2}, 10 min, NPs+NIR). Bright green signal representing the efficient generation of reactive oxygen species (ROS) is demonstrated from the cells treated with PI-$Au@Ag_2Se$-FA and NPs+NIR, whilst no ROS is seen from the untreated cells (Control) or the cells treated with $Au@Ag_2Se$-FA (Scale bar = 50 μm). All experiments are conducted at least twice independently, yielding similar results.

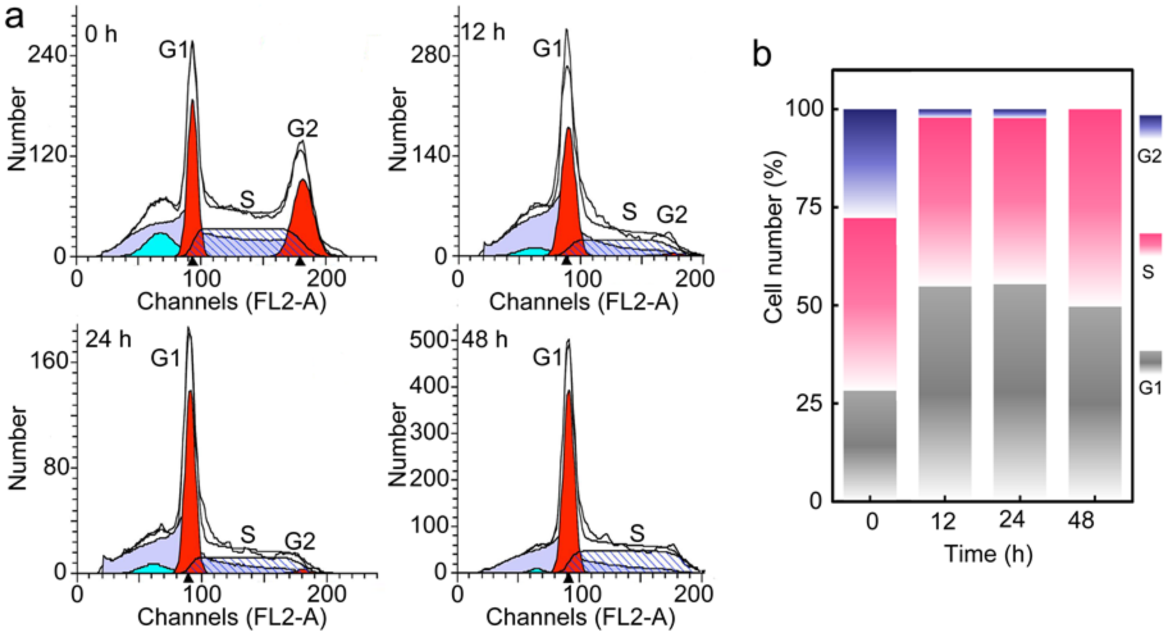

Supplementary Fig. 14 | a, Cell cycles and **b,** flow cytometric analysis of 4T1 cells incubated with PI-Au@Ag_2Se-FA (100 µg·mL^{-1} Au@Ag_2Se-FA pre-irradiated by 808 nm with a power density of 0.8 W·cm^{-2} for 10 min) for different duration. The increased G1 and decreased G2 of 4T1 cells indicate the disrupted cell cycles and effective chemotherapeutic effect of PI-Au@Ag_2Se-FA. All experiments are conducted at least twice independently, yielding similar results.

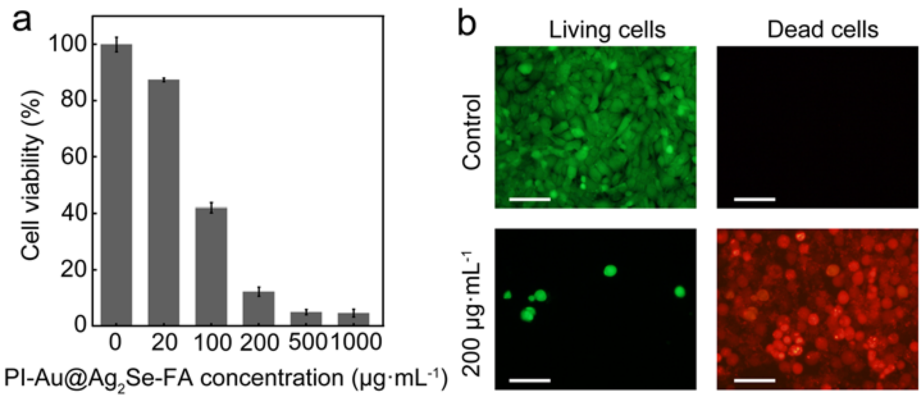

Supplementary Fig. 15 | a, Cell viability of 4T1 cells after 24h incubation with PI-Au@Ag_2Se-FA dispersions at various concentrations. **b,** Fluorescence images of 4T1 cells after 24 h incubation with PI-Au@Ag_2Se-FA (200 μg·mL^{-1}, scale bar = 25 μm). Whilst only 4.2% of 4T1 cells are killed by the pristine Au@Ag_2Se-FA (200 μg·mL^{-1}) without irradiation, 87% of 4T1 cells are killed by PI-Au@Ag_2Se-FA, indicating the critical contribution of chemodynamic therapy and chemotherapy of Ag nanocrystals/ions in the PI-Au@Ag_2Se-FA to the inhibition of cancer cells. All experiments are conducted at least twice independently, yielding similar results.

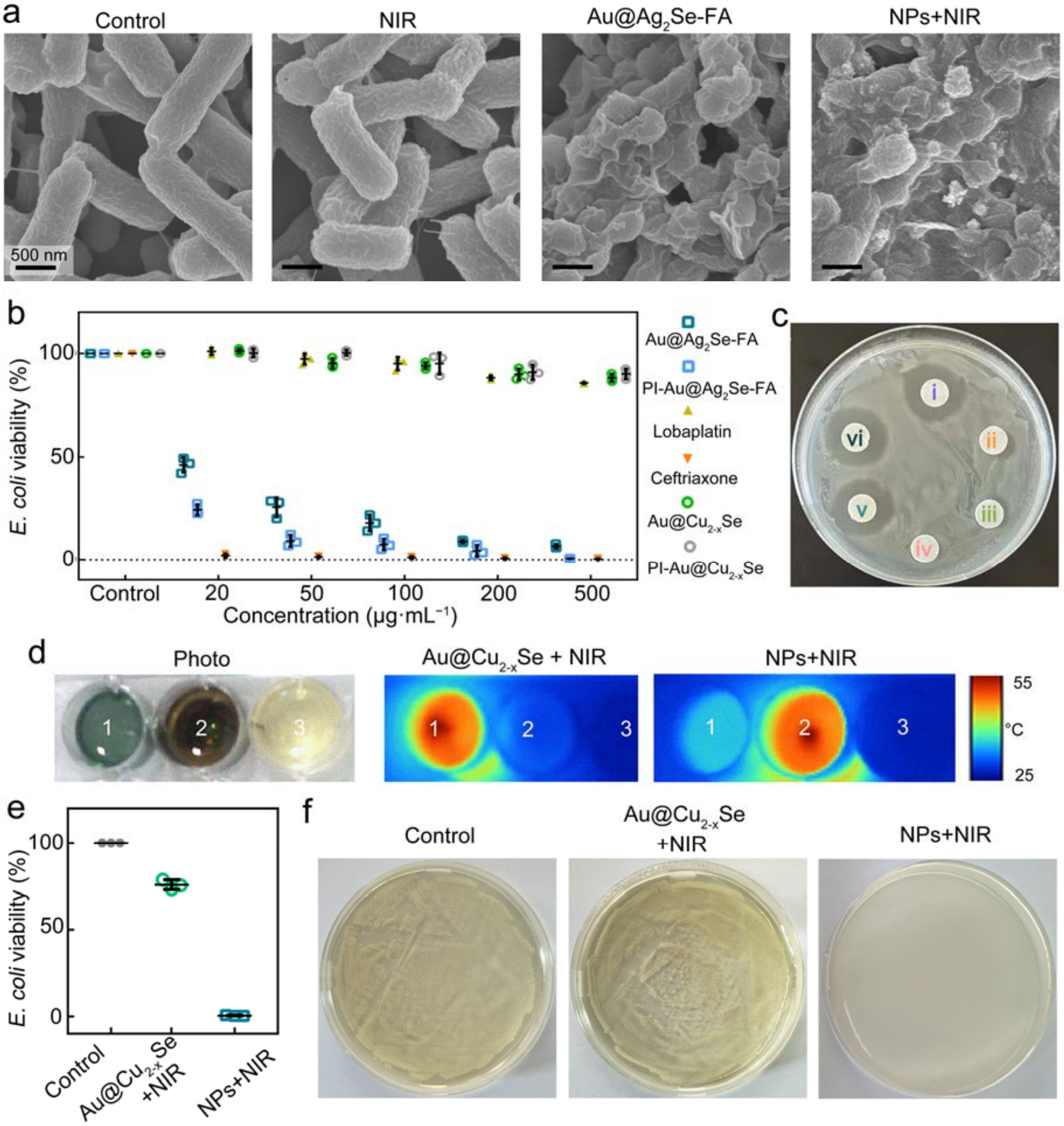

Supplementary Fig. 16 | a, *In vitro* antibacterial effect on *E. coil* by i) PBS only (Control), ii) 808 nm irradiation (0.8 W·cm^{-2}, 10 min) (NIR), iii) 24 h-incubation with Au@Ag_2Se-FA (100 μg·mL^{-1}) at 37 °C (Au@Ag_2Se-FA), and iv) Au@Ag_2Se-FA (100 μg·mL^{-1}) plus 10-min NIR irradiation (NPs+NIR). **b,** *E. coil* viability and **c,** Antibacterial efficacy analysis after 24 h incubation with i) ceftriaxone (100 μg·mL^{-1}), ii) lobaplatin (100 μg·mL^{-1}), iii) Au@Cu_{2-x}Se (100 μg·mL^{-1}), iv) PI-Au@Cu_{2-x}Se (100 μg·mL^{-1}), v) PI-Au@Ag_2Se-FA (100 μg·mL^{-1}) and vi) Au@Ag_2Se-FA, with *E. coli* incubated with PBS as Control (n = 3 experimental replicates). **d,** Digital and IRT images of *E. coil* incubated with Au@Cu_{2-x}Se (Well #1) and Au@Ag_2Se-FA (Well #2) before and upon 10 min irradiation, where the dispersion concentration is 100 μg·mL^{-1}. **e,** *E. coli* viability (n = 3 experimental replicates) and **f,** digital photos of bacteria culture analysis after the bacteria are treated with Au@Cu_{2-x}Se and Au@Ag_2Se-FA dispersion (100 μg·mL^{-1}) upon NIR irradiation (0.8 W·cm^{-2}, 10 min), with the cells treated by PBS as Control.

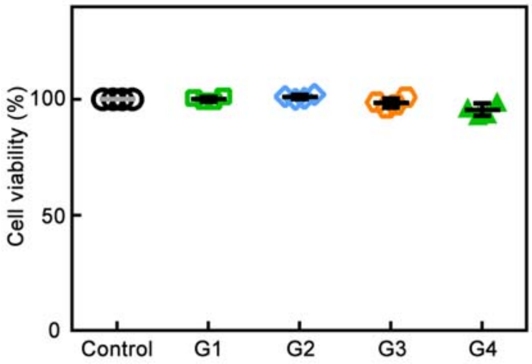

Supplementary Fig. 17 | Cell viability of M0 macrophages after treated with i) pristine Au@Ag_2Se-FA (100 μg·mL^{-1}) (G1), ii) PI-Au@Ag_2Se-FA (100 μg·mL^{-1}) (G2), iii) NIR irradiation only (0.8 W·cm^{-2}, 10 min) (G3), and iv) Au@Ag_2Se-FA plus NIR irradiation (G4), with the untreated M0 macrophage cells as Control (n = 4 experimental replicates).

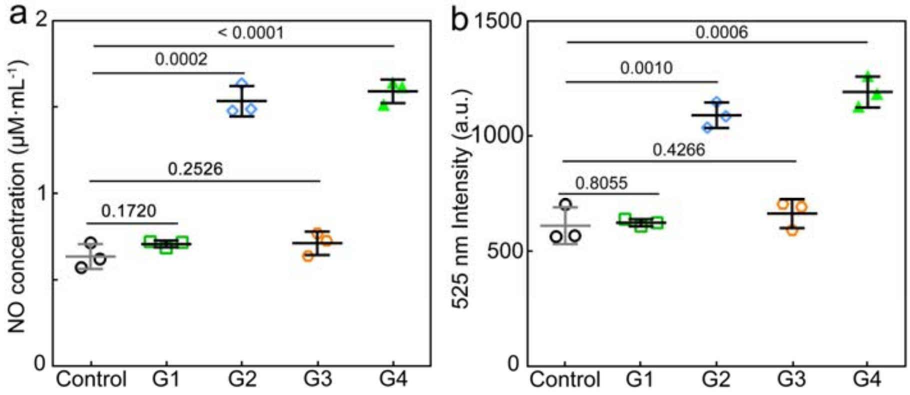

Supplementary Fig. 18 | a, Nitric oxide (NO) and **b,** ROS release from M0 macrophages by i) Au@Ag_2Se-FA (100 μg·mL^{-1}) without irradiation (G1), ii) PI-Au@Ag_2Se-FA (100 μg·mL^{-1}) (G2), iii) NIR irradiation only (0.8 W·cm^{-2}, 10 min) (G3), and iv) Au@Ag_2Se-FA plus NIR irradiation (G4) (n = 3 experimental replicates). Data are presented as mean ± standard deviation. One-way ANOVA with Tukey's post hoc test. $P < 0.05$ is considered to be statistically significant.

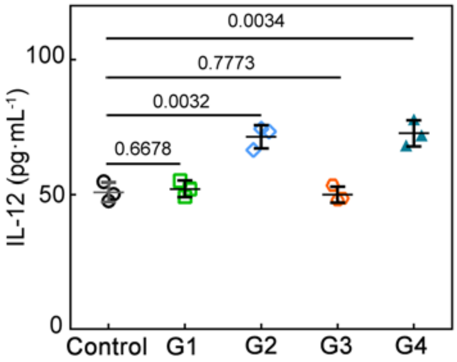

Supplementary Fig. 19 | IL-12 level in the supernatants from M0 macrophages treated with i) Au@Ag_2Se-FA (100 μg·mL^{-1}) (G1), ii) PI-Au@Ag_2Se-FA (100 μg·mL^{-1}) (G2), iii) NIR irradiation only (0.8 W·cm^{-2}, 10 min) (G3), and iv) Au@Ag_2Se-FA plus NIR irradiation (G4) (n = 3 experimental replicates). Data are presented as mean ± standard deviation. One-way ANOVA with Tukey's post hoc test. $P < 0.05$ is considered to be statistically significant.

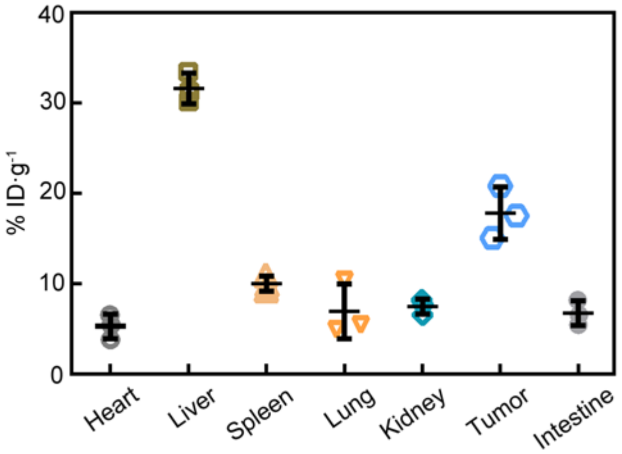

Supplementary Fig. 20 | Biodistribution of Au@Ag_2Se-FA in the major organs 12 h after *i.v.* injection, determined by the concentration of Au ions in the tissue lysates using ICP-MS (*n* = 3 mice per group).

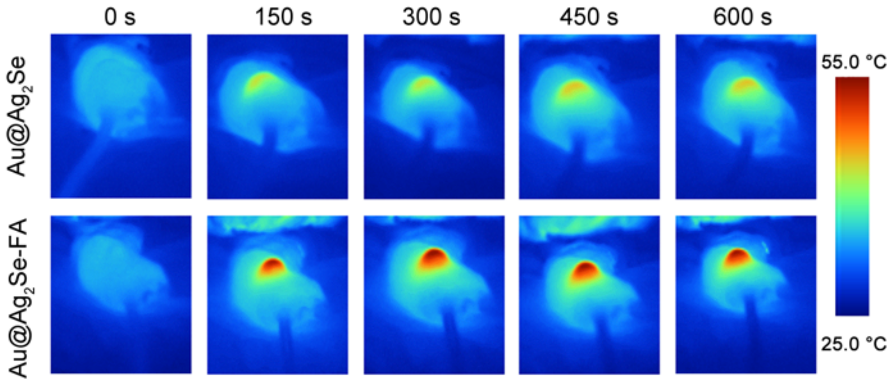

Supplementary Fig. 21 | *In vivo* IRT images of 4T1 tumor-bearing mice. Whilst the tumor temperature of mice treated by *i.v.* injection of Au@Ag_2Se is < 46 °C upon 10 min NIR irradiation (808 nm, 0.36 W·cm^{-2}), the tumor temperature of mice treated by *i.v.* injection of Au@Ag_2Se-FA quickly increases to ~55 °C upon the same irradiation, suggesting the enhanced tumor-targeting effect by FA modification. All experiments are conducted at least twice independently, yielding similar results.

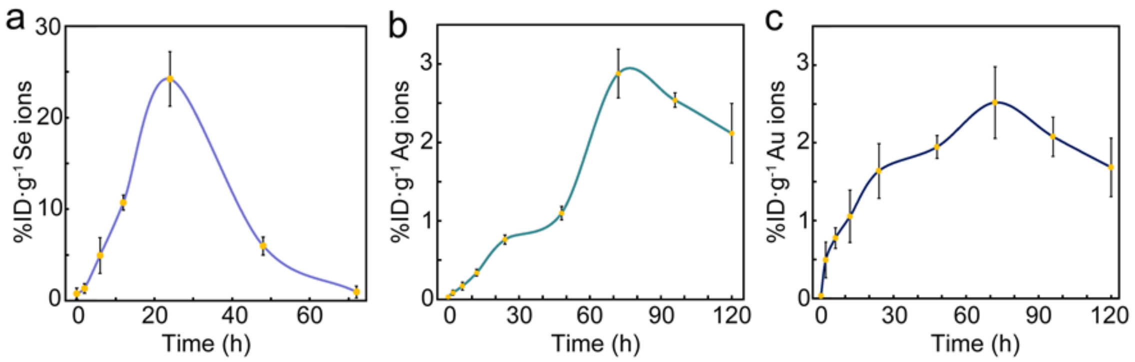

Supplementary Fig. 22 | Concentration of **a,** Se ions, **b,** Ag ions, and **c,** Au ions in urinary at different time points after *i.v.* injection of Au@Ag_2Se-FA dispersion (*n* = 3 mice per group). Data are presented as mean ± standard deviation. After injection, Balb/c mice were housed in metabolic cages. 4 h before each time point, their urine was collected, then digested using aqua regia, and measured using ICP-MS.

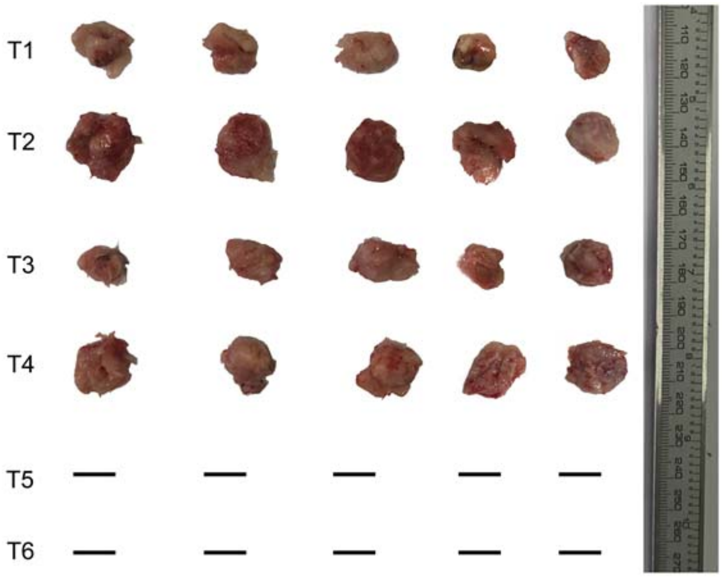

Supplementary Fig. 23 | Photos of the tumors collected from the mice 16 days after the various treatments (*n* = 5 mice per group).

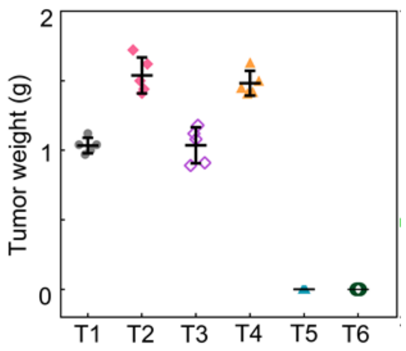

Supplementary Fig. 24 | Mean tumor weight collected from the mice 16 days after the various treatments (*n* = 5 mice per group). Data are presented as mean ± standard deviation.

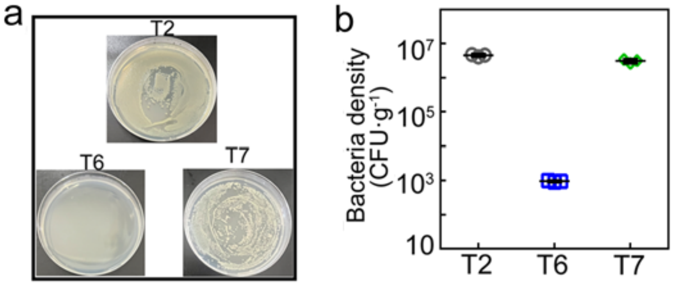

Supplementary Fig. 25 | a, Representative photos for bacteria culture analysis and **b,** bacteria density measured from the breast tumors of T2, T6, and T7 after various treatments (*n* = 3 mice per group).

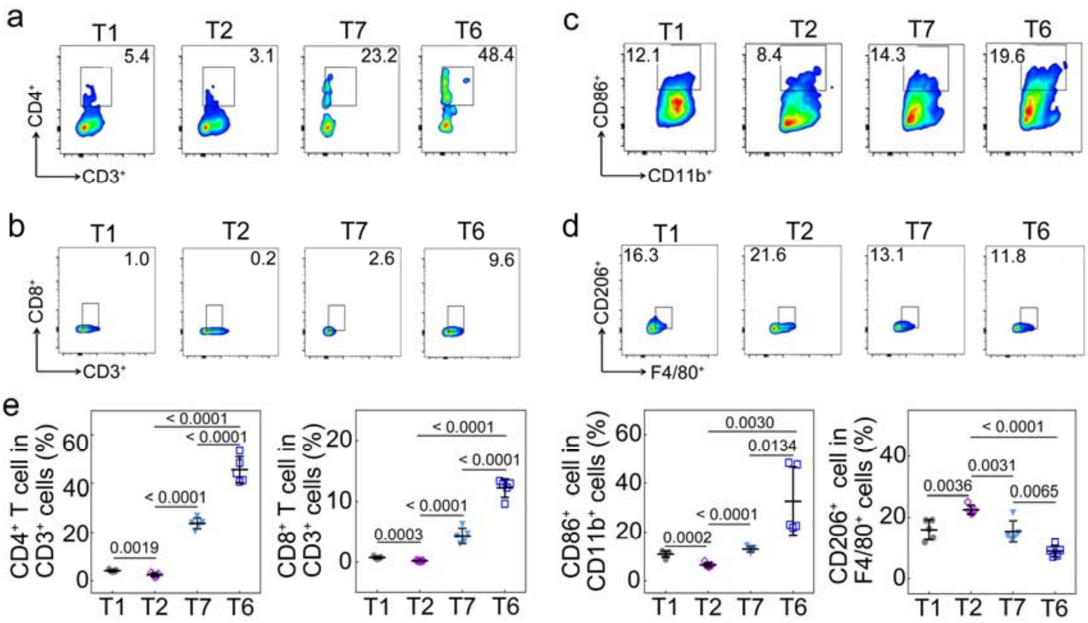

Supplementary Fig. 26 | a, Flow cytometric analysis of the helper ($CD3^+$, $CD4^+$) T cells, **b,** cytotoxic ($CD3^+$, $CD8^+$) T cells, **c,** M1-like ($CD86^+$, $CD11b^+$) and **d,** M2-like ($CD206^+$, $F4/80^+$) TAMs in the TME (n = 5 mice per group), and **e,** quantification of these cells after the various treatments, including i) PBS plus NIR irradiation (T1 and T2), ii) Au@Ag_2Se-FA plus irradiation (T6), iii) Au@Cu_{2-x}Se plus irradiation (T7), where the mice of T2, T6, and T7 were *i.t.* injected with *E. coli.* Data are presented as mean ± standard deviation. One-way ANOVA with Tukey's post hoc test. $P < 0.05$ is considered to be statistically significant.

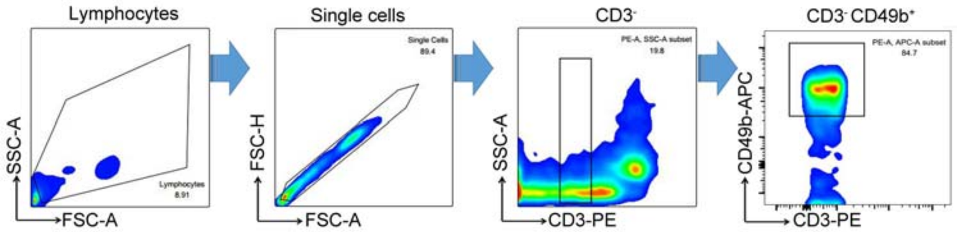

Supplementary Fig. 27| Representative flow cytometric gating strategy for NK cells (CD3^{-}, CD49b^{+}) panel in blood of the R-T5, R-T6, and control groups.

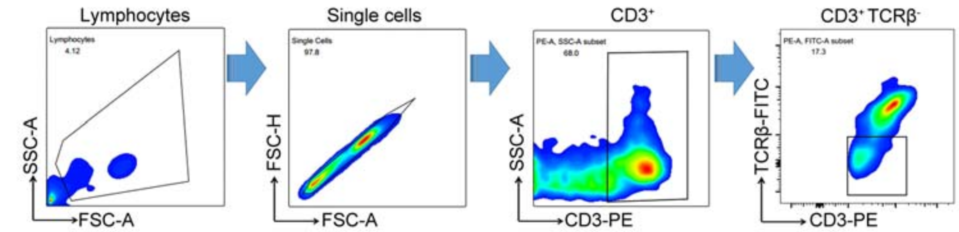

Supplementary Fig. 28 | Representative flow cytometric gating strategy for γδ T cells (CD3$^+$, TCRβ$^-$) panel in blood of the R-T5, R-T6, and control groups.

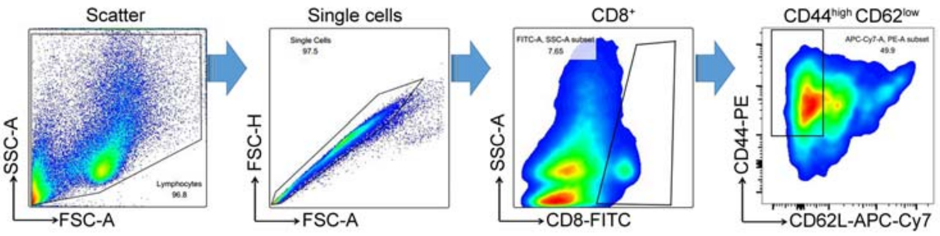

Supplementary Fig. 29 | Representative flow cytometric gating strategy for memory T cells (CD8^{+}, CD44^{high}, CD62L^{low}) panel in spleens of the R-T5, R-T6, and control groups.

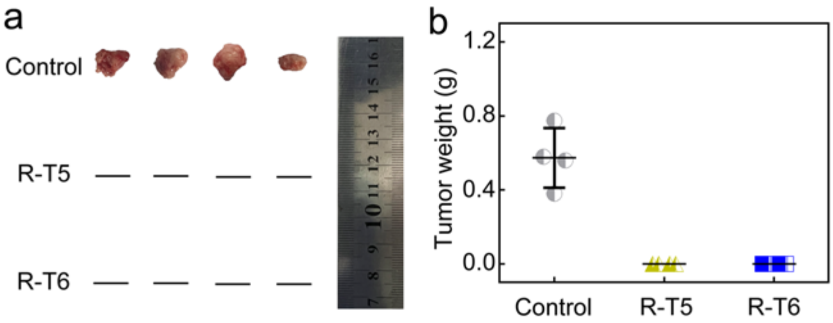

Supplementary Fig. 30 | a, Digital photo (tumors in Contol at Day 21) and **b,** mean weight of the tumors collected from the mice 21 days after the various treatments (*n* = 4 mice per group).

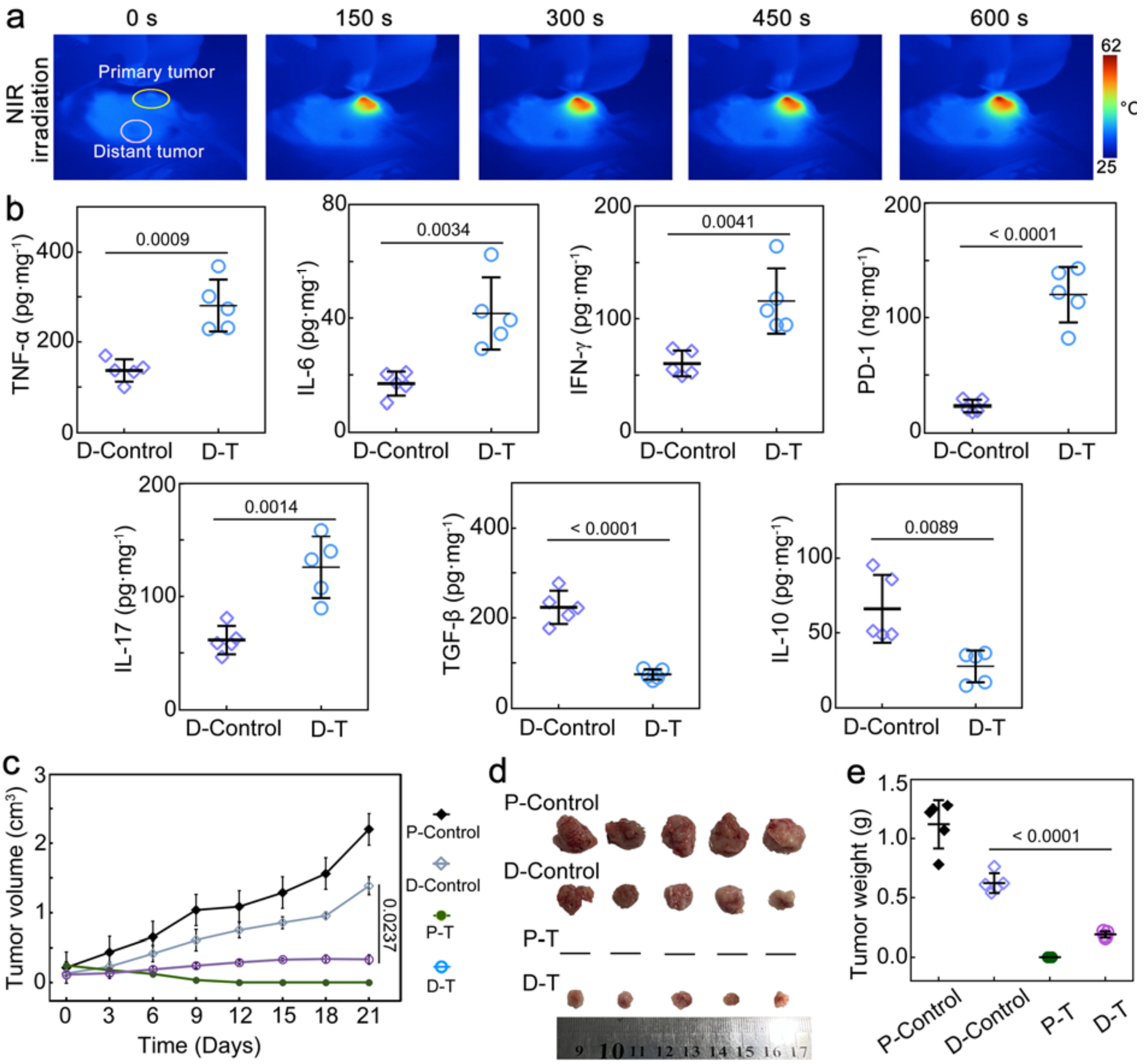

Supplementary Fig. 31 |*In vivo* therapeutic and immune effect on the distal tumor. Four different tumor groups are investigated, including i) the primary tumors treated with *i.t.* injection of Au@Ag_2Se-FA (50 μL, 3 mg·mL^{-1}) plus irradiation for 10 min (808 nm, 0.36 W·cm^{-2}) (P-T) and ii) their corresponding distal tumors (D-T), and iii) the primary tumors treated with *i.t.* injection of PBS (50 μL) plus irradiation for 10 min (808 nm, 0.36 W·cm^{-2}) (P-Control) and iv) their corresponding distal tumors (D-Control). Note that the distal tumors receive no treatment. **a,** *In vivo* IRT images of 4T1 tumor-bearing mice showing that the P-T group tumor reaches ~62°C, while the C-T group tumor temperature remains unchanged upon the 10 min irradiation. **b,** Intratumoral expression of TNF-α, IL-6, IFN-γ, PD-1, IL-17, TGF-β, and IL-10 for the D-T and D-Control groups (*n* = 5 mice per group). **c,** Tumor growth curves of the P-T, C-T, P-Control, and C-Control groups (*n* = 5 mice per group). **d,** Digital photos, and **e,** mean weight of the primary and distal tumors collected 21 days after the various treatments (*n* = 5 mice per group). Data are presented as mean ± standard deviation. One-way ANOVA with Tukey's post hoc test (**b**, **e**). Two-tailed Student's *t*-test (**c**). *P* < 0.05 is considered to be statistically significant.

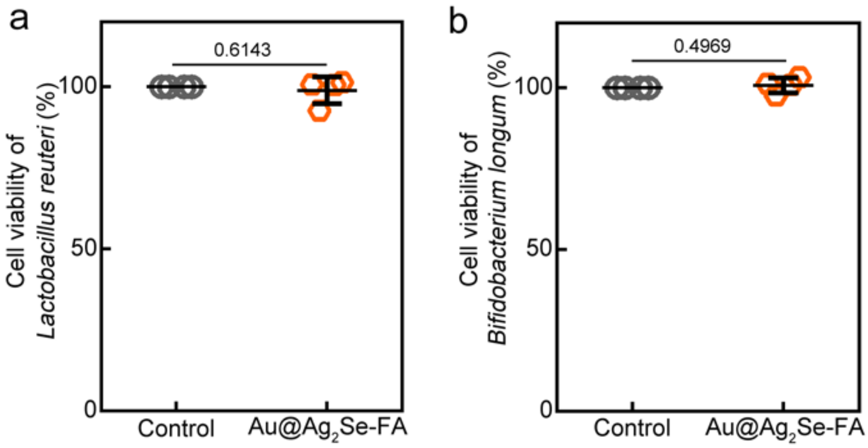

Supplementary Fig. 32 | Cell viability of **a,** *Lactobacillus reuteri* and **b,** *Bifidobacterium longum* suspension at 600 nm after co-incubating the bacteria with Au@Ag_2Se-FA (100 μg·mL^{-1}) or PBS (Control) (n = 4 experimental replicates). One-way ANOVA with Tukey's post hoc test. $P < 0.05$ is considered to be statistically significant.

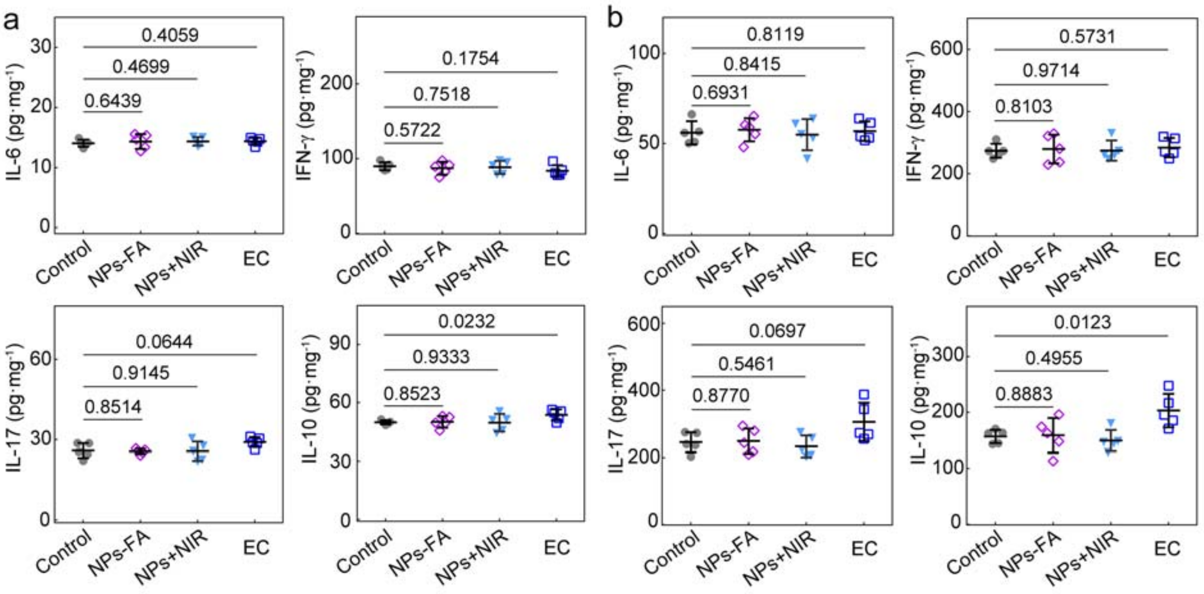

Supplementary Fig. 33| Cytokine concentration of IL-6, IFN-γ, IL-17, and IL-10 in **a,** serum and **b,** inguinal lymph nodes of healthy mice treated with i) *i.v.* injection of Au@Ag_2Se-FA (NPs) ii) *i.v.* injection of Au@Ag_2Se-FA plus irradiation (NPs+NIR), and iii) intradermally injected with *E. coli* (EC) (*n* = 5 mice per group), with untreated healthy mice as the control. There is no difference between the NPs and NPs+NIR groups compared to Control, indicating that Au@Ag_2Se-FA or the combination of Au@Ag_2Se-FA and irradiation cannot up-regulate or down-regulate the key immune cytokines (IL-6, IFN-γ, IL-17, and IL-10) of healthy mice. Data are presented as mean ± standard deviation. One-way ANOVA with Tukey's post hoc test. $P < 0.05$ is considered to be statistically significant.

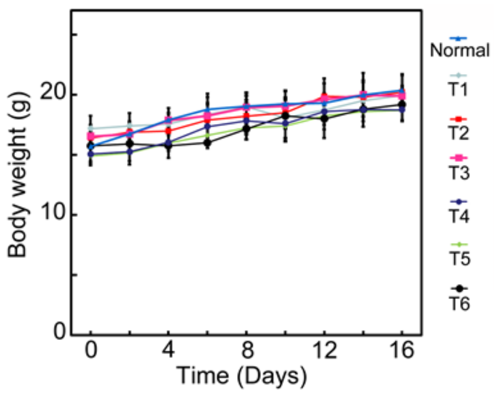

Supplementary Fig. 34 | Body weight of mice in different groups after the various treatments (n = 5 mice per group). Data are presented as mean ± standard deviation.

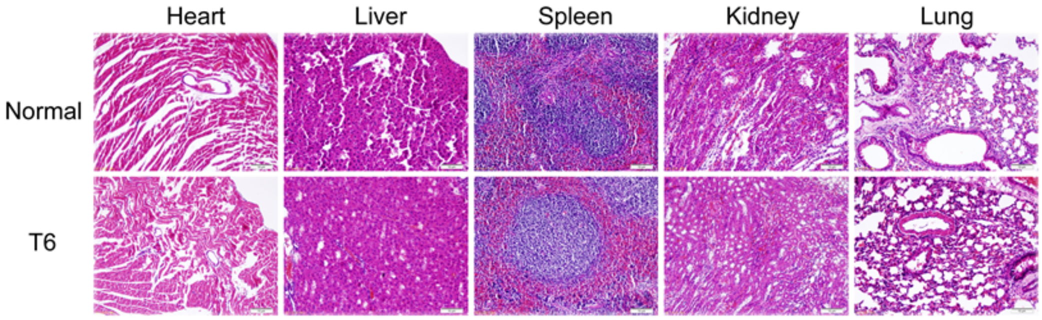

Supplementary Fig. 35 | Hematoxylin and eosin analysis of the major organs collected from healthy mice (Normal) and T6 group. Compared with Control, no noticeable inflammation or organ damage is presented from all the major organs in T6 (Scale bar = 50 μm).

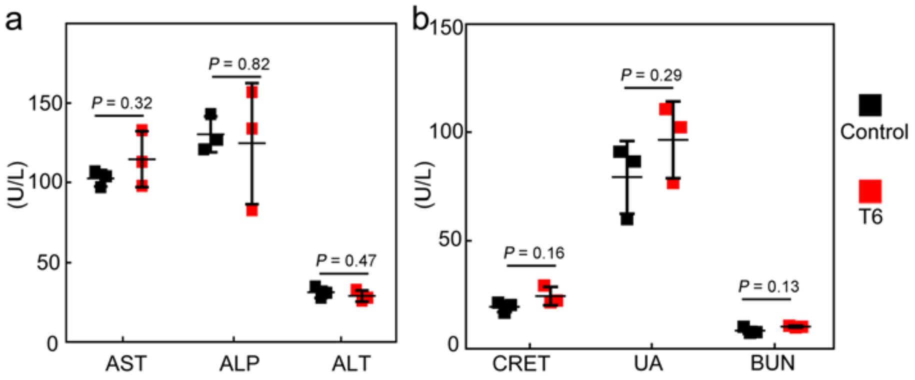

Supplementary Fig. 36| Serum biochemistry analysis of the mice treated by NPs+NIR (T6) for **a,** liver function markers including alkaline phosphatase (ALP), aspartate aminotransferase (AST), and alanine aminotransferase (ALT), and **b,** renal function markers including creatinine (CREA, μmol·mL^{-1}), uric acid (UA, μmol·mL^{-1}), and blood urea nitrogen (BUN, mmol·mL^{-1}) (n = 3 mice per group). The values of liver function markers and renal function markers of T6 are close to those of healthy mice (Normal). $P < 0.05$ is considered to be statistically significant.

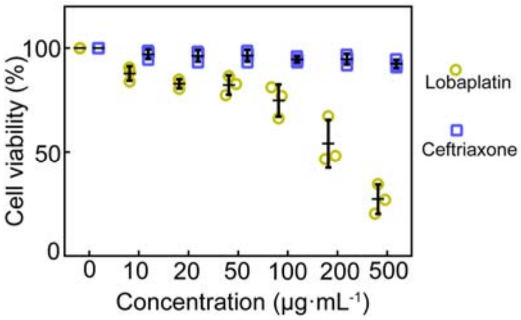

Supplementary Fig. 37 | Cell viability of 4T1 cells after 24 h incubation with lobaplatin (alone) and ceftriaxone (alone) at various concentrations (*n* = 3 experimental replicates). Lobaplatin shows strong cytotoxicity against 4T1 cells as a chemotherapeutics, whilst ceftriaxone shows no noticeable cytotoxicity.

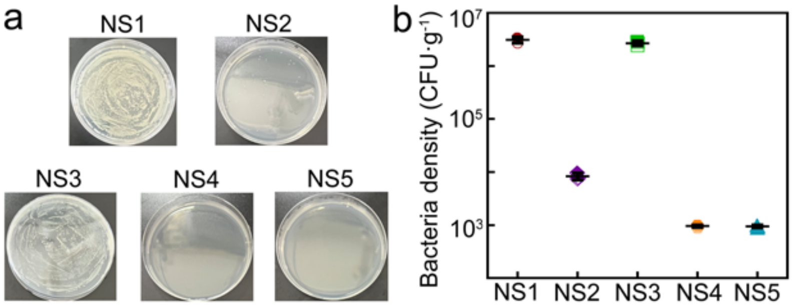

Supplementary Fig. 38 | a, Representative photos for bacteria culture analysis, and **b,** bacteria density measured from the breast tumors of the NS1, NS2, NS3, NS4, and NS5 groups after the different treatments (*n* = 3 mice per group).

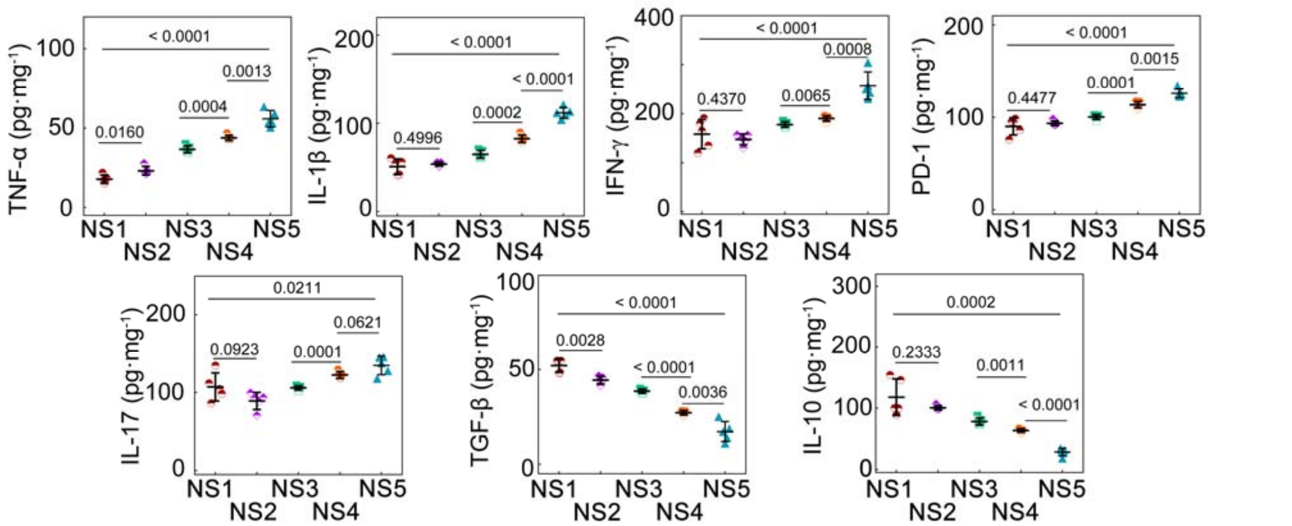

Supplementary Fig. 39 | Intratumoral expression level of TNF-α, IL-1β, IFN-γ, PD-1, IL-17, TGF-β and IL-10 for the tumors of mice raised in a non-sterile environment. The mice were divided into five groups (n = 5 mice per group), including i) the untreated group (NS1), the mice treated ii) by ceftriaxone alone (NS2), iii) by lobaplatin alone (NS3), iv) by the combination of ceftriaxone and lobaplatin (NS4), and v) by Au@Ag_2Se-FA plus irradiation (NS5). Data are presented as mean ± standard deviation. One-way ANOVA with Tukey's post hoc test. $P < 0.05$ is considered to be statistically significant.

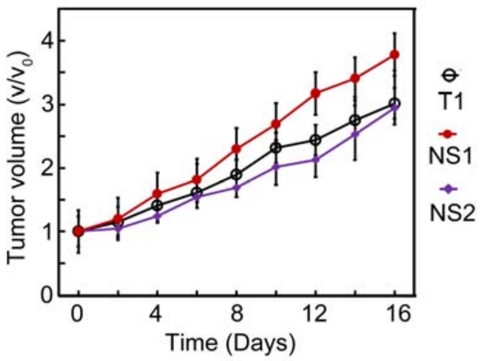

Supplementary Fig. 40 | Tumor growth rate of the T1, NS1 and NS2 groups. The tumor-bearing mice of NS1 and NS2 are raised in a non-sterile environment with total bacteria of 7500 CFU/m^3; the mice of T1 are raised in a sterile environment (n = 5 mice per group).

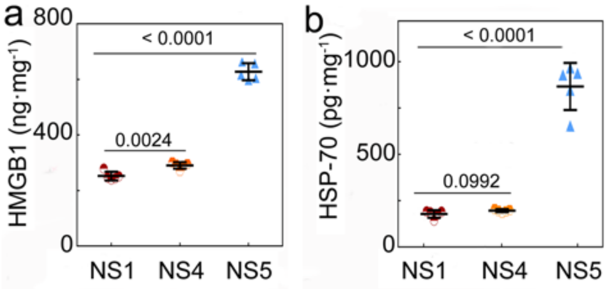

Supplementary Fig. 41 | a, High mobility group box1 (HMGB1) and **b,** heat shock protein (HSP)-70 levels in the tumors of the mice *i.t.* injected with ceftriaxone + lobaplatin (500 μg·mL^{-1}, 50 μL, NS4) and those *i.v.* injected with Au@Ag_2Se-FA (10 mg·mL^{-1}, 100 μL, NS5) plus NIR irradiation (0.36 W·cm^{-2}, 10 min), with the untreated tumor-bearing mice as Control (NS1) (n = 5 mice per group). Data are presented as mean ± standard deviation. One-way ANOVA with Tukey's post hoc test. $P < 0.05$ is considered to be statistically significant.

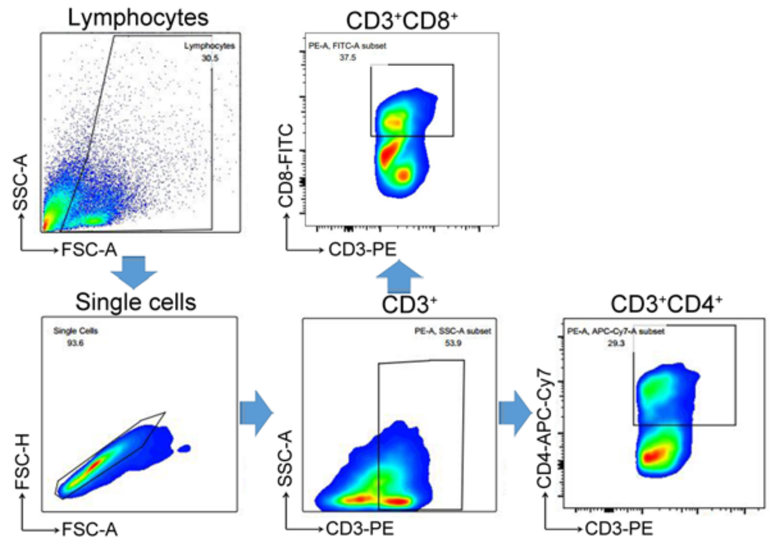

Supplementary Fig. 42 | Representative flow cytometric gating strategy for help ($CD3^+$, $CD4^+$) and cytotoxic ($CD3^+$, $CD8^+$) T cells panel in the tumors of the RM-C, RM-T2, RM-T3, and RM-T4 groups three days after the various treatments. The RM-T2 and RM-T4 groups were treated by *i.t.* injection of *E. coli* three days before the treatments. RM-T2 had no subsequent treatment. RM-T3 and RM-T4 were treated by NIR irradiation 12 h after *i.v.* injection of Au@Ag_2Se-FA. RM-C was the control group without any treatment.

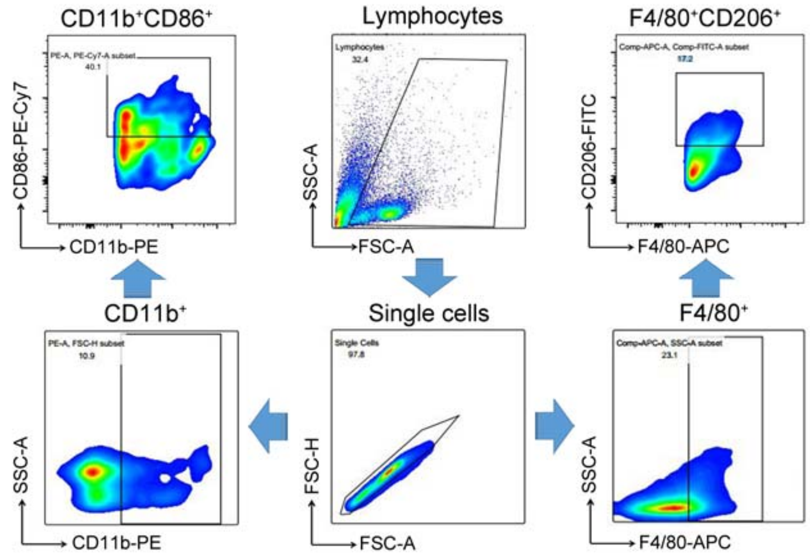

Supplementary Fig. 43 | Representative flow cytometric gating strategy for M1-like ($CD86^+$, $CD11b^+$) and M2-like ($CD206^+$, $F4/80^+$) TAMs panel in the tumors of the RM-C, RM-T2, RM-T3, and RM-T4 groups three days after the various treatments.

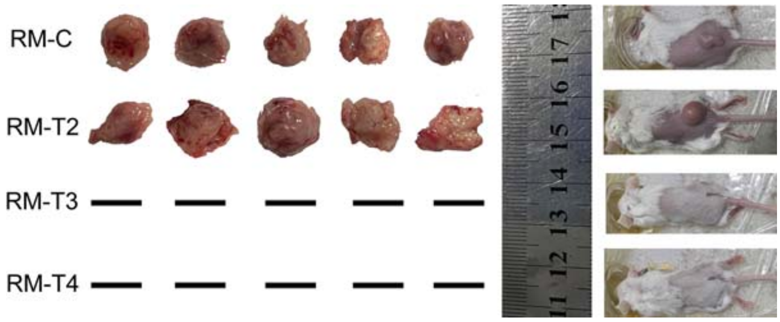

Supplementary Fig. 44 | Digital photos of the mice and tumors (collected from the mice) 24 days after the various treatments (*n* = 5 mice per group).

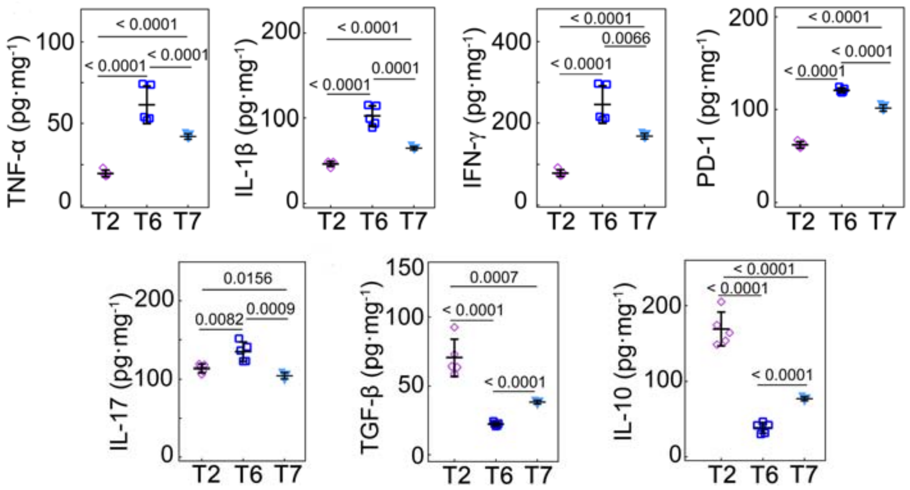

Supplementary Fig. 45 | Intratumoral expression level of TNF-α, IL-1β, IFN-γ, PD-1, IL-17, TGF-β, and IL-10 for the tumor-bearing mice *i.t.* injected with *E. coli* upon the different treatments (n = 5 mice per group), including i) PBS plus NIR irradiation (T2), ii) Au@Ag_2Se-FA plus irradiation (T6) and iii) Au@Cu_{2-x}Se plus irradiation (T7). Data are presented as mean ± standard deviation. One-way ANOVA with Tukey's post hoc test. $P < 0.05$ is considered to be statistically significant.

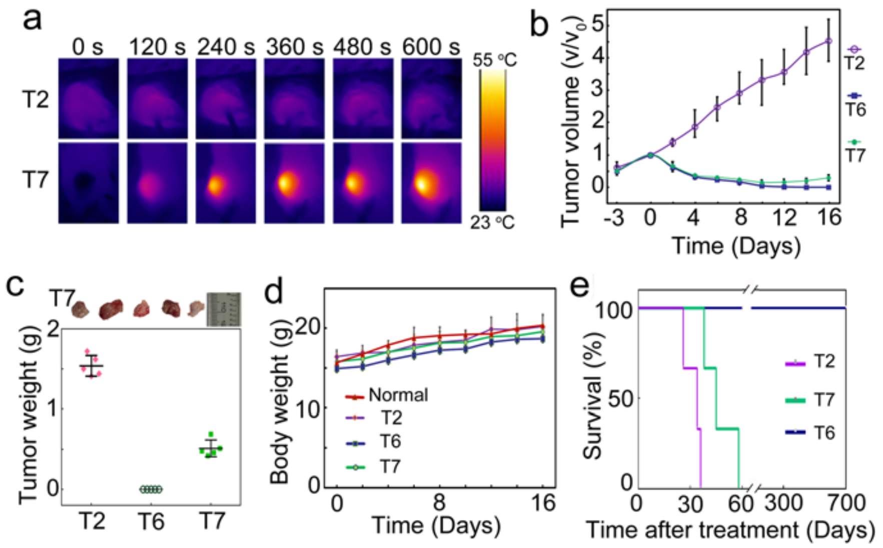

Supplementary Fig. 46| **a,** *In vivo* IRT images of 4T1 tumor-bearing mice: whilst the tumor temperature of T2 (treated with PBS injection) is < 40°C upon 10 min NIR irradiation (808 nm, 0.36 W·cm^{-2}), the tumor temperature of T7 (treated with *i.t.* injection of Au@Cu_{2-x}Se NPs) quickly climbed to ~55 °C upon the same irradiation. **b,** Tumor growth rate in the different groups (*n* = 5 mice per group): all groups receive an *i.t.* injection of *E. coli* followed by one of three treatments: i) *i.v.* injection of PBS plus NIR irradiation (T2), ii) *i.v.* injection of Au@Ag_2Se-FA plus NIR irradiation (T6), or iii) *i.t.* injection of Au@Cu_{2-x}Se plus NIR irradiation (T7). The irradiation conditions are 808 nm, 0.36 W·cm^{-2}, and 10 min. **c,** Digital photos (tumors in T7 on Day 16 post-treatment) and mean tumor weight from the mice at 16 days after the various treatments (*n* = 5 mice per group). **d,** Body weight of mice in the different groups after the various treatments (*n* = 5 mice per group). **e,** Survival curves after the various treatments (*n* = 5 mice per group). All mice in T7 are dead within 58 days after treatment. Data are presented as mean ± standard deviation.

Supplementary Table 1 | Calculation details for photothermal conversion efficiency of Au@Ag_2Se-FA.

h and *S* are the heat transfer coefficient and the surface area of the sample container; T_{max} and T_{surr} are the equilibrium temperature and ambient temperature of the surroundings; Q_s is the baseline energy input by the solvent and the sample container without Au@Ag_2Se-FA; *I* is the laser power; and A_λ is the absorbance of the sample at 808 nm.

hS	16.034 mW·°C^{-1}
T_{max}-Ts_{urr}	24.2 °C
Q_s	10.416 mW
A_λ	0.27
I	1200 mW
η	68.0 %

Supplementary Table 2 | Primer sequences marker genes of M1-like and M2-like TAMs for quantitative real-time polymerase chain reaction analysis.

Gene	NCBI Accession NO.		Primer 5'-3'	Product size (bp)
HsTnf-α	NM_000594.4	Forward	CCTCTCTCTAATCAGCCCTCTG	220
		Reverse	GAGGACCTGGGAGTAGATGAG	
HsArg-1	NM_001244438.2	Forward	ACGGAAGAATCAGCCTGGTG	116
		Reverse	ATCAGTGTGAGCATCCACCC	
HsFizz-1	NM_032579.3	Forward	CGTCCTCTTGCCTCCTTCTC	227
		Reverse	ACAAGCACAGCCAGTGACAG	
Actb	NM_001101.5	Forward	GGACTTCGAGCAAGAGATGG	234
		Reverse	AGCACTGTGTTGGCGTACAG	
