# Supplementary material for: Long-term relapse-free survival enabled by integrating targeted antibacteria in antitumor treatment
Source: Nat Commun. 2024 May 17;15:4194. doi: 10.1038/s41467-024-48662-x (PMC11101653; doi:10.1038/s41467-024-48662-x)
Supplement: Supplementary file 3 — Reporting Summary [file 41467_2024_48662_MOESM3_ESM.pdf]

nature portfolio

Corresponding author(s): Miao Yu

Last updated by author(s): Mar 12, 2024

Reporting Summary

Nature Portfolio wishes to improve the reproducibility of the work that we publish. This form provides structure for consistency and transparency in reporting. For further information on Nature Portfolio policies, see our Editorial Policies and the Editorial Policy Checklist.

Statistics

For all statistical analyses, confirm that the following items are present in the figure legend, table legend, main text, or Methods section.

n/a	Confirmed	
☐	☒	The exact sample size (*n*) for each experimental group/condition, given as a discrete number and unit of measurement
☐	☒	A statement on whether measurements were taken from distinct samples or whether the same sample was measured repeatedly
☐	☒	The statistical test(s) used AND whether they are one- or two-sided<br>*Only common tests should be described solely by name; describe more complex techniques in the Methods section.*
☒	☐	A description of all covariates tested
☐	☒	A description of any assumptions or corrections, such as tests of normality and adjustment for multiple comparisons
☐	☒	A full description of the statistical parameters including central tendency (e.g. means) or other basic estimates (e.g. regression coefficient) AND variation (e.g. standard deviation) or associated estimates of uncertainty (e.g. confidence intervals)
☐	☒	For null hypothesis testing, the test statistic (e.g. *F*, *t*, *r*) with confidence intervals, effect sizes, degrees of freedom and *P* value noted<br>*Give P values as exact values whenever suitable.*
☒	☐	For Bayesian analysis, information on the choice of priors and Markov chain Monte Carlo settings
☒	☐	For hierarchical and complex designs, identification of the appropriate level for tests and full reporting of outcomes
☒	☐	Estimates of effect sizes (e.g. Cohen's *d*, Pearson's *r*), indicating how they were calculated

Our web collection on statistics for biologists contains articles on many of the points above.

Software and code

Policy information about availability of computer code

Data collection	Infrared thermal image software (Ti25, Fluke, USA); No custom code was used for data collection.
Data analysis	All statistical analysis was performed using Graphpad Prism (v6.0). All flow-cytometry data were analyzed on FlowJo software (v10; BDBiosciences, USA). Image J (1.8) was used to analyze the immunohistochemical results.

For manuscripts utilizing custom algorithms or software that are central to the research but not yet described in published literature, software must be made available to editors and reviewers. We strongly encourage code deposition in a community repository (e.g. GitHub). See the Nature Portfolio guidelines for submitting code & software for further information.

Data

Policy information about availability of data

All manuscripts must include a data availability statement. This statement should provide the following information, where applicable:

- Accession codes, unique identifiers, or web links for publicly available datasets
- A description of any restrictions on data availability
- For clinical datasets or third party data, please ensure that the statement adheres to our policy

The authors declare that all data supporting the findings of this study are available within the paper [and its Supplementary Information]. Source data are provided with this paper.

Research involving human participants, their data, or biological material

Policy information about studies with human participants or human data. See also policy information about sex, gender (identity/presentation), and sexual orientation and race, ethnicity and racism.

Reporting on sex and gender	*Use the terms sex (biological attribute) and gender (shaped by social and cultural circumstances) carefully in order to avoid confusing both terms. Indicate if findings apply to only one sex or gender; describe whether sex and gender were considered in study design; whether sex and/or gender was determined based on self-reporting or assigned and methods used. Provide in the source data disaggregated sex and gender data, where this information has been collected, and if consent has been obtained for sharing of individual-level data; provide overall numbers in this Reporting Summary. Please state if this information has not been collected. Report sex- and gender-based analyses where performed, justify reasons for lack of sex- and gender-based analysis.*
Reporting on race, ethnicity, or other socially relevant groupings	*Please specify the socially constructed or socially relevant categorization variable(s) used in your manuscript and explain why they were used. Please note that such variables should not be used as proxies for other socially constructed/relevant variables (for example, race or ethnicity should not be used as a proxy for socioeconomic status). Provide clear definitions of the relevant terms used, how they were provided (by the participants/respondents, the researchers, or third parties), and the method(s) used to classify people into the different categories (e.g. self-report, census or administrative data, social media data, etc.) Please provide details about how you controlled for confounding variables in your analyses.*
Population characteristics	*Describe the covariate-relevant population characteristics of the human research participants (e.g. age, genotypic information, past and current diagnosis and treatment categories). If you filled out the behavioural & social sciences study design questions and have nothing to add here, write "See above."*
Recruitment	*Describe how participants were recruited. Outline any potential self-selection bias or other biases that may be present and how these are likely to impact results.*
Ethics oversight	*Identify the organization(s) that approved the study protocol.*

Note that full information on the approval of the study protocol must also be provided in the manuscript.

Field-specific reporting

Please select the one below that is the best fit for your research. If you are not sure, read the appropriate sections before making your selection.

☒ Life sciences ☐ Behavioural & social sciences ☐ Ecological, evolutionary & environmental sciences

For a reference copy of the document with all sections, see nature.com/documents/nr-reporting-summary-flat.pdf

Life sciences study design

All studies must disclose on these points even when the disclosure is negative.

Sample size	Sample sizes were determined by G-power analysis software to the One-way ANOVA with Tukey's post hoc test.
Data exclusions	No data were excluded.
Replication	The experiments were conducted at least twice independently, yielding consistent results. Replicates were reproducible.
Randomization	All biological experiments were randomized.
Blinding	Blinding was used in all biological experiments.

Reporting for specific materials, systems and methods

We require information from authors about some types of materials, experimental systems and methods used in many studies. Here, indicate whether each material, system or method listed is relevant to your study. If you are not sure if a list item applies to your research, read the appropriate section before selecting a response.

Materials & experimental systems

n/a	Involved in the study
☐	☒ Antibodies
☐	☒ Eukaryotic cell lines
☒	☐ Palaeontology and archaeology
☐	☒ Animals and other organisms
☒	☐ Clinical data
☒	☐ Dual use research of concern
☒	☐ Plants

Methods

n/a	Involved in the study
☒	☐ ChIP-seq
☐	☒ Flow cytometry
☒	☐ MRI-based neuroimaging

Antibodies

Antibodies used	For flow cytometry, CD3-PE (clone: 17A2), CD4-APC-cy7 (clone: GK1.5), CD8a-FITC (clone: 53-6.7), CD11b-PE (clone: M1/70), F4/80-APC (clone: BM8), CD86-PE-Cy7 (clone: GL-1), CD206-FITC (clone: C068C2), CD49b-APC (clone: HMα2), TCRβ-FITC (clone: H57-597), CD44-PE (clone: IM7), and CD62L-APC-Cy7 (clone: MEL-14) were purchased from Biolegend. Anti-CD3 (Ablonal, A1753, dilution 1:100), anti-CD86 (Ablonal, A2352, dilution 1:100), anti-ARG-1 (Ablonal, A4923, dilution 1:100), and secondary antibodies (HRP Goat anti-rabbit IgG, Ablonal, AS014, dilution 1:100) were employed for IHC analysis.
Validation	The supplier validated all antibodies and tested each lot for quality. These antibodies were used without further validation.

Eukaryotic cell lines

Policy information about cell lines and Sex and Gender in Research

Cell line source(s)	4T1, RM-1, HUVEC, and Tohoku Hospital Pediatrics-1 (THP-1) cells (human acute monocytic leukemia cell line) were purchased from National Collection of Authenticated Cell Cultures (Shanghai, China).
Authentication	The cells lines were authenticated by Short Tandem Repeat (STR) profiling.
Mycoplasma contamination	All cell lines were tested for mycoplasma contamination. No mycoplasma contamination was found.
Commonly misidentified lines (See ICLAC register)	No commonly misidentified cell lines were used.

Animals and other research organisms

Policy information about studies involving animals; ARRIVE guidelines recommended for reporting animal research, and Sex and Gender in Research

Laboratory animals	Female and male BALB/c mice (4–6 weeks) were purchased from Beijing Vital River Laboratory Animal Technology Co., Ltd.
Wild animals	The study did not involve wild animals.
Reporting on sex	The study did not involve the reporting on sex.
Field-collected samples	The study did not involve samples collected from field.
Ethics oversight	All animal experiments were carried out under protocols approved by the Institutional Animal Care and Use Committee of Harbin Institute of Technology. The approval number is IACUC-2019021.

Note that full information on the approval of the study protocol must also be provided in the manuscript.

Flow Cytometry

Plots

Confirm that:

☒ The axis labels state the marker and fluorochrome used (e.g. CD4-FITC).

☒ The axis scales are clearly visible. Include numbers along axes only for bottom left plot of group (a 'group' is an analysis of identical markers).

☒ All plots are contour plots with outliers or pseudocolor plots.

☒ A numerical value for number of cells or percentage (with statistics) is provided.

Methodology

Sample preparation	Tissue samples: the tumor tissues were first mechanically disrupted from mice, divided into small pieces, and homogenizedin dissociation buffer [mixture of deoxyribonuclease (50 μg·mL–1), hyaluronidase (100 μg·mL–1) and collagenase IV (2mg·mL–1)] at 37 °C for 1 h with gentle shaking. The cell suspension was passed through a 70 μm cell strainer and dispersedin phosphate buffered saline to form the single-cell suspension, and then stained with the indicated antibodies.
Instrument	BD LSRFortessa (BD Biosciences)
Software	FlowJo (v10; BD Biosciences, USA)
Cell population abundance	N/A
Gating strategy	Cells were first gated on FSC/SSC, then gated on the basis of positive and fluorescence minus one controls,and the frequencies of the cells staining positive for each marker was recorded.

☒ Tick this box to confirm that a figure exemplifying the gating strategy is provided in the Supplementary Information.
